# Evaluation of surface electromyography of selected neck muscles during the whiplash mechanism in aware and unaware conditions due to safe punching in kickboxing

**DOI:** 10.1186/s12891-023-06563-y

**Published:** 2023-05-30

**Authors:** Mosa Pashaei, Farideh Babakhani, Kambiz Banihashemi

**Affiliations:** Department of Sport Injuries and Corrective Exercises, Faculty of Physical education and sport science, Allameh Tabataba’i University, Tehran, Iran

**Keywords:** Combat sport of Kickboxing, Surface electromyography, Whiplash injuries, Whiplash mechanism

## Abstract

**Background:**

Kickboxing is considered as a combat sport in progress, in which injuries are frequent and significant, and close injury monitoring is highly recommended. Sports injuries to the head and neck are estimated to cause 70% deaths and 20% permanent disabilities although they are much less common than those to the limbs. Whiplash mechanism involves the rapid extension (opening) and flexion (bending) of neck. The purpose of the current study was to investigate the electromyographic activity of selected muscles in the whiplash mechanism in aware and unaware conditions of the safe punching in kickboxing so that we can design special exercises.

**Method:**

In the present study, 24 male kickboxing athletes aged 18–40 years were selected based on a purposive sampling method. The surface electromyography (EMG) signals of muscles were recorded with and without awareness of safe punching by using a nine-channel wireless EMG device. Additionally, a nine-channel 3D inertial measurement unit (IMU, wireless,) was utilized to determine the acceleration, kinematics, and angular velocity of the subjects’ head. The statistical dependent t-test was applied to compare the EMG activity of each muscle, as well as its participation ratio.

**Results:**

The results of statistical analysis represented a significant increase in the EMG activity of sternocleidomastoid (p = 0.001), upper trapezius (p = 0.001) and cervical erector spinae muscles (p = 0.001), as well as the neck extension and flexion angles between the athletes aware (open eyes) and unaware (closed eyes) of the safe punching.

**Conclusion:**

In this study, the EMG activity of the sternocleidomastoid, upper trapezius, and cervical erector spine muscles in the aware condition was significantly different from the activity under unaware condition. In fact, the intended muscles exhibited significantly different behaviors in preventing extension and flexion in the two conditions.

## Background

Kickboxing is considered as a relatively young sport originated from karate. The structure of this sport involves elements of karate, taekwondo, and boxing [1]. In this combat sport, frequent and significant injuries occur, the close monitoring on which is strongly recommended. The lack of high-quality epidemiological data on kickboxing, especially about injury severity, suggests an urgent need for further research [2]. It is estimated that 70% of deaths and 20% of permanent disabilities are caused by sports injuries to the head and neck despite their lower rate compared to that of injuries to limbs [3]. In addition, neck whiplash is an extension-flexion motion of the neck. It was previously assumed that muscles do not contribute to the injury [4]. The mechanism of the injury includes the rapid extension (opening) of the neck, followed by its rapid flexion (bending). The whiplash injury has been reported in the traffic accidents in which a vehicle is hit from behind by another. In the collisions while driving, the trunk, head, and neck of individuals simultaneously move forward due to inertia, and this flexion often plays no role in whiplash injury. Then, the neck and head quickly go into hyperextension and rapidly return to flexion in order, where the two motions cause injury [5]. Given that the cervical spine is prone to injury in whiplash, it is less compatible when resisting inertial loading [6]. Further, whiplash is caused by a sudden acceleration-deceleration mechanism which transfers energy to the cervical spine [7]. The neck pain and dysfunction, stiff neck, headache, dizziness, visual impairment, psychological distress, and memory, concentration, and temporomandibular disorders can be addressed as some of the complications in this injury [8]. The other consequences are muscle weakness, fatigue, and atrophy, as well as cervical vertebral osteoarthritis, anxiety, radicular symptoms, disorder in sleep and brainstem cranial nerves [9], shoulder pain, and upper-limb numbness [7]. The earlier pain, further symptoms, and more initial disability are associated with the slower recovery [10]. A study of mixed martial arts (MMA) analyzed by video camera kinematics of neck movements concluded that the risk of whiplash injury in MMA is significant and there are no safety regulations to address these concerns [11]. Due to the few studies on the whiplash mechanism in sports, accidents are used to explain the issue. In another study, eight healthy males were seated in a sled seat, and the whiplash mechanism accelerated by a spring mechanism was simulated. Then, the surface electromayography (EMG) of their sternocleidomastoid (SCM) and upper trapezius (UT) muscles was measured at various accelerations. The results revealed that muscles could affect the injury pattern. In fact, the flexor muscle (sternocleidomastoid) reached peak magnitude fast enough to be within the time(52.9 ms) of head acceleration. It is noteworthy that clinically symptoms are often attributed to muscle tendon in juries. It can be speculated that these injuries occur from negative or eccentric muscle contractions due to the lag between motion and peak muscle activity. Thus, the muscle involvement can as well be a disadvantage in the whiplash injury mechanism. There were no differences between expected and unexpected conditions [4]. Siegmund et al. exposed the individuals warned and unwarned about the whiplash mechanism to the mechanism in a rear accident and examined the EMG of their paraspinal and SCM muscles. They reported 7 ms earlier activation of the muscle response in the warned condition compared to the unwarned condition [12]. Based on the results of another study, the EMG of neck SCM and hyoid muscles in the whiplash mechanism was 49% faster and 80% greater among aware individuals than the unaware ones [13]. Homayunpour et al. determined the EMG of SCM and UP muscles in 18 males and females warned about whiplash mechanism and were subjected to automatic emergency braking. Compared to the unaware subjects, the aware ones exhibited significantly different muscle responses based on gender and age [14]. Kumar et al. reported that the response of SCM muscles, especially trapezius, is greater at higher acceleration in the frontal impacts by analyzing low-velocity frontal collosions using EMG and kinematics [15]. Despite the general belief that whiplash injuries mainly occur in rear-end collisions, the results of some crash studies and frontal impact sports have been implicated as the cause of a large number of whiplash injuries [15, 16]. In a study of 53 male and female rugby players to record head impact events, the isometric strength of their neck muscles was measured. Muscle activity in uncontrolled whiplash mechanism was lower in women than in men. There is an increase in the EMG activity of the SCM muscle with an increase in the magnitude of the impact, and there are differences between the EMG of the SCM muscle of men and women [16]. Heikkila and Wenngren found no relationship between joint position sense and pain intensity, and consequently the dysfunction in proprioceptive system among whiplash injury patients [17]. So far, few studies have assessed the EMG activity of neck muscles, and no comprehensive information is available in this regard. Thus, it seems necessary to perform a study to evaluate the EMG activity of Sternocleidomastoid (SCM), Uper Trapezius (UT), and cervical erector spinae (ES) muscles, as well as examining the neck extension and flexion angles following the whiplash mechanism caused by a safe punching in kickboxing. The purpose of the current study was to investigate the electromyographic activity of selected muscles in the whiplash mechanism in aware and unaware conditions of the safe punching in kickboxing so that we can design special exercises.

## Methods

### Participants

The present study was conducted among 24 eligible male kickboxing athletes of 18–40 years old in Qiamdasht city, the number of which was obtained by G-POWER 3.1 software [14]. All measurements were conducted in the Fightland Martial Club located in Qiamdasht city (Ray County, Tehran Province) under the same environmental conditions. The inclusion criteria were the age range of 18–40 years, BMI of 18.5–29.9, and lack of any injury in the shoulder girdle and neck joints, as well as practicing kickboxing exercises at least three times a week continuously. Any complication and inability to perform motion, BMI more than 30 (obesity), upper-limb muscle injury related to the past six months, and musculoskeletal deformities in the neck and shoulder girdle region were defined as the exclusion criteria [14].

Prior to the test, ethical approval was obtained by the ethical committee of Allameh Tabataba’i university, and all participants provided written informed consent Also, additional informed consent was obtained from all individual participants for whom identifying information is included in this article.

## Procedure

**Fig. 1:**
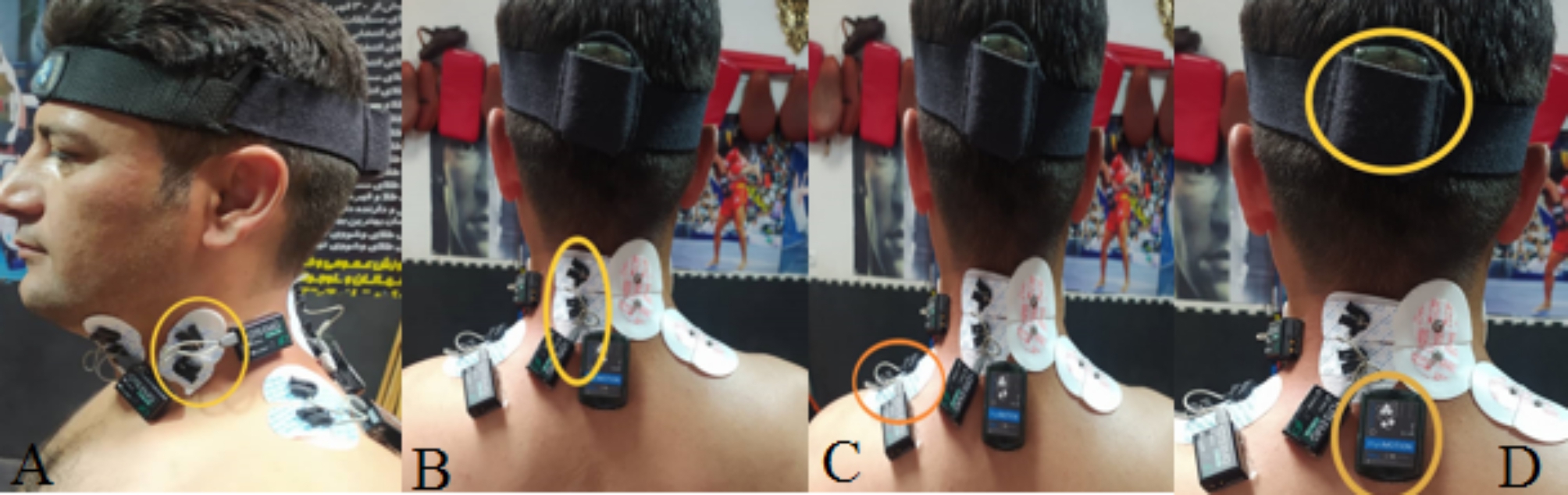
Placing EMG electrodes (SCM: Fig **A**, ES: Fig **B**, UP: Fig **C**) and mounting IMU sensor (Fig **D**)

A nine-channel wireless EMG device was applied to determine the EMG activity of SCM, UT, and cervical erector spinae (ES) muscles. Additionally, the acceleration of safe punching, as well as neck extension and flexion angles, and kinematics of the athletes were recorded on a nine-channel 3D motion capture inertial measurement unit (IMU). A world champion hit the safe punching, the mean velocity of which was obtained by using an IMU device (3306˚/s) (two sensors were installed in the arm and forearm). He was selected since he could control the punching at the mentioned velocity during the test due to high experience in championship and coaching. Disposable silver chloride surface electrodes (SKINTACT) were used in the electrode placement stage. Further, the skin surface was first shaved and sanded to reduce skin resistance and increase the quality of muscle signals. The electrodes were placed with the center-to-center distance of 30 mm along the muscle fibers based on the bipolar method and approach presented in the previous studies [18]. The EMG activity of SCM as a representative of neck flexors), cervical ES muscles (as a representative of neck extensors), and UT muscles were determined during the safe punching. Regarding the SCM muscle, the electrode was located on the most outstanding area of its sternal head at the lower third of the beginning and end of the muscle (mastoid process to sternal notch) [19]. The electrode of the cervical ES was positioned on the surface of the fourth cervical vertebra at a distance of 1.5 cm external to the spine process [15]. In the UT muscle, the electrode was placed on the half-line from the acromion to the spine in the seventh cervical vertebra (C7) [20](Fig. 1). To measure neck extension and flexion during safe punching, two IMU sensors with an elastic band and special adhesive were placed on the back of the head and the C7, and its frequency was set to (sampling rate 100 Hz). [31].(Fig. 1 ).

**Fig. 2:**
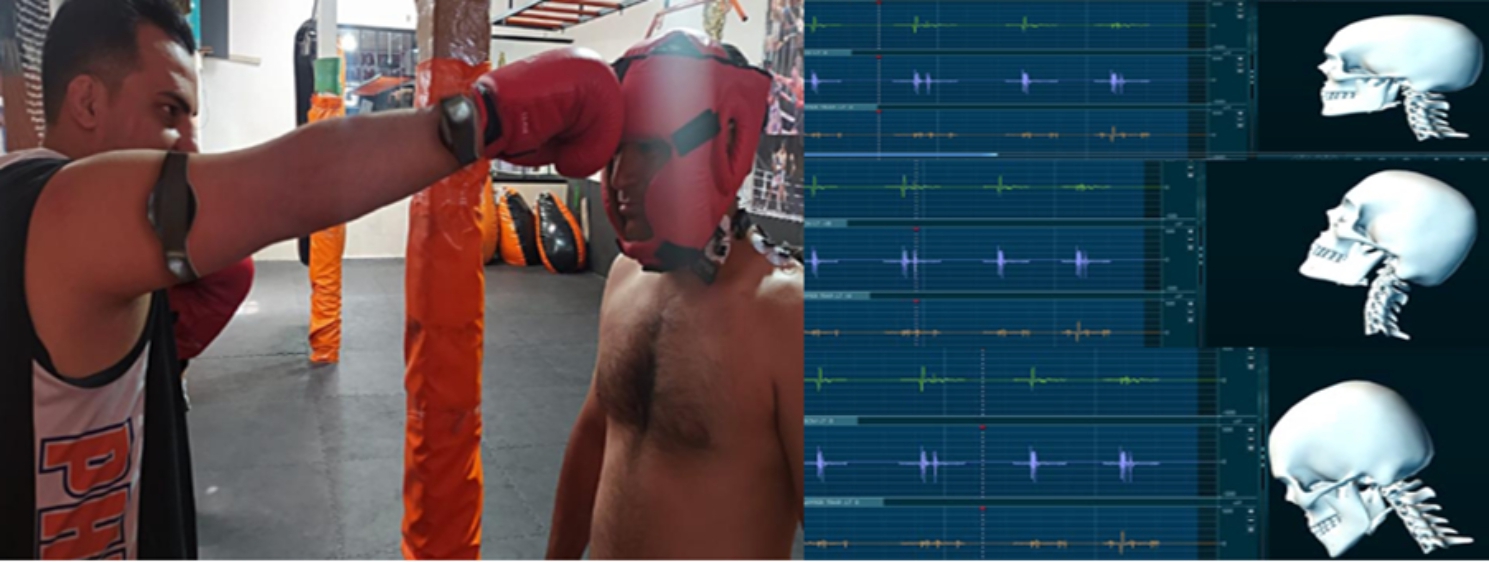
Measuring the EMG of the muscles, and the neck extension and flexion angles

The next stage involved evaluating the EMG activity of the selected muscles, as well as the neck extension and flexion angles. To this end, the athletes were prepared with the relevant equipment and stood while their eyes were open (in aware conditions), three safe punching were hit on their foreheads by the world champion, who had two IMU sensors installed in his arm and forearm (Fig. 2). A 10-second rest was given after each punching. Then, three safe punching were delivered on the forehead when the eyes were closed (unaware of the impact). It is worth noting that the subjects were warned while contracting muscles in the unawarned conditions, and the punching was applied while the muscles were relaxed. In this test, the muscle activity was calculated 0.5 s before (onset) and 1.5 s after the punching (offset) so that a total of two seconds was examined for each test. The EMG signals were recorded at the sampling frequency of 1500 Hz, and were filtered by the 4th order Butterworth high-pass filter with cut off frequency of 10 Hz, detrended, fully-wave rectified, and then low-pass filtered at 30 Hz. The onset and offset of the muscles were determined based on the maximum muscle activity during the activity. The equipment used for athletes includes gloves and boxing caps (top ten) and a safe blow to the forehead is used to prevent injury.

### Statistical analysis

Raw signals were assessed using MATLAB software, and statistical analysis was performed in SPSS 25 software (Microsoft Corp., Redmond, WA). The Shapiro-Wilk test was utilized to check the conformity of the data with the normal numerical distribution, so that the dependent t-test was used to analyze the difference between the two conditions. Finally, tables and diagrams were provided by using Excel software (Microsoft Corp., Redmond, WA).

## Results

The results of the correlated t-test (paired) indicated that the activity of each of the SCM, UT, and cervical ES muscles was statistically significant under aware and unaware conditions in the onset (p = 0.001), offset (p = 0.001), and total activity (2 s) (p = 0.001). The results of the neck extension angle with open eyes (p = 0.001) and neck flexion angle with closed eyes were significant (p = 0.001) (Table 1).

**Table 1:** T-test results for comparing the EMG of the SCM, UT, and ER muscles in aware and unaware conditions (onset/offset refers to with/without awareness of the safe punching), as well as the results of the neck extension and flexion angles^^^

Variable	Aware condition M $\pm$ SD	Unaware condition M $\pm$ SD	High and low confidence Interval	Correlation	p.value
SCM onset (ms)	2.98 $\pm$ 1.81	1.97$\pm 1.19$	1.30—0.71	0.988	< 0.001^*^
SCM offset (ms)	5.79 $\pm$ 2.09	4.01$\pm 1.53$	2.23—1.32	0.871	0.001^*^
UT onset (ms)	3.68$\pm 1$	2.53$\pm 1.01$	1.5—0.79	0.659	< 0.001^*^
UT offset (ms)	4.97$\pm 1.36$	3.76$\pm 1.09$	1.58—0.82	0.75	< 0.001^*^
ER onset (ms)	6.91$\pm 1.94$	4.62$\pm 1.54$	2.8—1.78	0.784	< 0.001^*^
ER offset (ms)	8.67$\pm 1.89$	6.85$\pm 1.55$	2.1—1.53	0.964	0.001^*^
Extension angle (°)	6.39$\pm 1.73$	29.24 $\pm$1.98	(-21.7) — (-24)	-0.058	< 0.001^*^
Flexion angle (°)	29.03$\pm 3.1$	42.49$\pm 3.9$	(-11.25) — (-15.67)	-0.078	0.001^*^

^^^Data are presented as mean ± SD. ^*^Significant level: *p* ≤ 0.05

## Discussion

The present study focused on the EMG activity of SCM, UT, and ES muscles in the whiplash mechanism in aware and unaware conditions of the safe punching in kickboxing. In this regard, the two tests of safe punching in aware and unaware conditions were designed, the results of which specified the main difference in the activity of the selected muscles in the above-mentioned conditions. The results demonstrated a significant difference between the normalized EMG activity of the SCM muscle in the two conditions (P < 0.05). In other words, a significant difference was found between the subjects aware and unaware of the punching in terms of the EMG activity of the muscle at the onset (0.5 s before the safe punching), offset (1.5 s after the impact), and total activity (2 s). In addition, there was a limitation in collecting the information of these muscles (ES) due to the interference in the neck area. The results are consistent with those of Homayunpour which represented a significantly higher EMG activity of SCM muscle among the subjects sitting in sled who were warned about applying a 20-N force from the back under controlled conditions to induce the whiplash mechanism than the unwarned ones [13]. Additionally, muscle injuries, especially the stretches caused by contraction, are a function of the pre-activation, strong stretching, and initial length of muscles, all of which are related to the loss of contractile force. Regarding a certain pressure, a rise in muscle activation level may lead to more muscle injury, the severity of which is determined based on the amount of applied force [21]. The role of the aware will is not to initiate a specific volitional act, but to select and control the outcome of the volition. The aware will can act in a permissive manner, or allow or prevent the movement of an action intention that arises uncawerly. Alternatively, there may be a need for an aware activation or stimulation without which the final motor output will not follow the brain’s unaware priming and preparation processes [22]. Some studies have reported a neuromechanical delay between the neck muscle activation and head motion in the incidents which may cause a whiplash mechanism such as car accident and head trauma during sports [4, 12, 23, 24]. The greater intensity and extent of impact, as well as shorter stimulus time can be addressed as the factors enhancing the EMG amplitude [25]. Awareness of an impending event alters the injury risk and kinematic response of head during a car collision or sports impact [12, 26–28]. Kumar et al. examined 10 healthy individuals in a sled at various accelerations with pneumatic cylinder impacts, some of whom were warned of the frontal collision to the sled, while the others were unaware [15]. They declared that the EMG activity of SCM and ES muscles was less than 30% in the unaware,while a twice activity was observed under the aware condition. However, the EMG activity of UT muscle reached a maximum value of 38–79%. Higher acceleration improves muscle activity. Further, the bone preparation of the head and cervical spine can bear only 1.4–1.5 time of head weight [15]. Neck muscles probably play the main role in causing injury [29], and possible stretch reflexes are modulated by muscle spindles, Golgi tendon organ, or both. Therefore, establishing the exact injury mechanism may help with primary prevention and corrective measures [15]. The results of the present study revealed that the normalized EMG activities of the UT muscle with awareness of the punching were significantly different from its level in the unaware condition (P < 0.05). In fact, the subjects aware and unaware of safe punching had a significantly different muscle EMG activity at the onset, offset, and total activity, which is line with the results of Kumar et al. [15] and Santos et al. [14]. Santos et al. subjected the volunteers seated inside a bus to an emergency braking test at 15 km/h under aware and unaware conditions. The EMG activity of the UT and SCM muscles was measured at the beginning and end of braking, the results of which indicated a significant difference in the muscle EMG activity of those warned and unwarned about the braking. Furthermore, females exhibited a significantly greater muscle activity than the males. It seems that the last stage (post-braking causing a whiplash mechanism) has a higher risk of neck muscle injury, which may be attributed to the continuity of the motion of passengers’ neck after stopping the vehicle (experiencing rebound), leading to an overload on the neck, which changes between the performances of the UT and SCM muscles until stopping the motion of the head relative to the trunk. Among the aware individuals, the neck muscles tightness decreases relative motion with respect to the trunk and can reduce the risk of cervical vertebral injury [14]. Based on the results of the ES muscle in the present study, a significant difference was detected between the normalized EMG activity of the athletes aware and unaware of the punching in kickboxing (P < 0.05). In other words, the aware and unaware conditions led to a significant difference in the muscle EMG activity at the onset, offset, and total activity, which is in consistent with the result of Brault et al. [30]. They exposed the individuals seated in a sled to rear collision at given velocities and reported early onset EMG activity of neck extensor muscle during the kinematic response of the head and trunk compared to the SCM one. In addition, the muscle EMG activity significantly changes by varying the velocity. The initial rearward retraction of the head relative to the trunk results in lengthening the SCM muscle, causing rapid neck muscle contraction in response to the impact and injury due to over-lengthening. The muscle length stretched before stimulation affects its force generated during stimulation. In the case of a muscle stretched more than its rest length, force declines since sarcomeres become so long that the myosin cross-bridges cannot reach the junctions on the thin filaments and participate in the contraction. However, the results of the present study and Kumar et al. [15] are not in line with those of Siegmund et al. [12]. Siegmund et al. induced a whiplash mechanism among the subjects sat in a car seat mounted on a sled in aware, unaware, and unexpected conditions by applying a controlled force from the back to the seat. The results suggested no significant difference in the neck extensor and SCM muscles of the aware and unaware individuals. The lack of significant difference may be related to the relatively slow velocity of test to avoid the injury of the subjects, as well as not adjusting the sitting position by the warned and unwarned ones. Notably, crosstalk might have occurred between these muscles due to using surface electrodes, although this is a well-known limitation of this widely utilized methodology. Fanta O. announced in a research Kinematic values indicate more favourable parameters for neck injuries for visual. Head injury criteria show an average decrease of about 30% for visual. they concluded that the visual perception means a significant increase in pre-activation of the observed muscle group of almost 400% and lower activation in both following phases of approximately 40%. awareness and an active preparation in advance through increasing motor neuron potential helps to reduce head deceleration and therefore it is possible to expect less severe injury. It was proven that expecting the impact increases neck muscle activation by 400%. This activa- tion culminates approx. 130 ms before impact in the case the upcoming impact is recognized. This results in lower head deceleration course and head displacement angle and consequently in decrease of maximum head deceleration and head injury criterion (35).Due to the limitations of this topic, few studies have been done in sports. In order to prevent common injuries in the neck, it is expected that studies will be directed towards sports so that we can reduce the injuries caused to athletes.

## Conclusion

In the present study, real tests were conducted in the kickboxing club by hitting safe punching (velocity: 3306˚/s). Compared to the unaware condition, the SCM muscle, as a representative of the neck flexors, was recruited with more activity in the aware conditions, the activity of which significantly influenced the prevention of neck extension. Further, a significant difference was observed between the warned and unwarned subjects in terms of the neck extension, reflecting the risk factor for neck injury in unaware conditions. Regarding the UT and cervical ES muscles, the activities were significantly different in the conditions. Furthermore, they prevented the neck flexion although the flexion was relatively high in among unaware individuals, indicating the risk factor for neck injury in both conditions (Extension aware and unaware:6.39 and 29.24)(Flexion aware and unaware:29.03 and 42.49).But in an unaware conditions, flexion and neck extension increases, and this causes damage in the posterior and anterior regions of the neck. .We hope that by understanding the results of muscle activity in two conditions (aware and unaware), we can encourage coaches to implement neuromuscular coordination and proprioception exercises to restrain and reduce risk of the neck injuries with the whiplash mechanism in kickboxing; So that we can reduce some of the worries of this sport.

## Data Availability

The datasets used and/or analysed during the current study available from the corresponding author on reasonable request.
